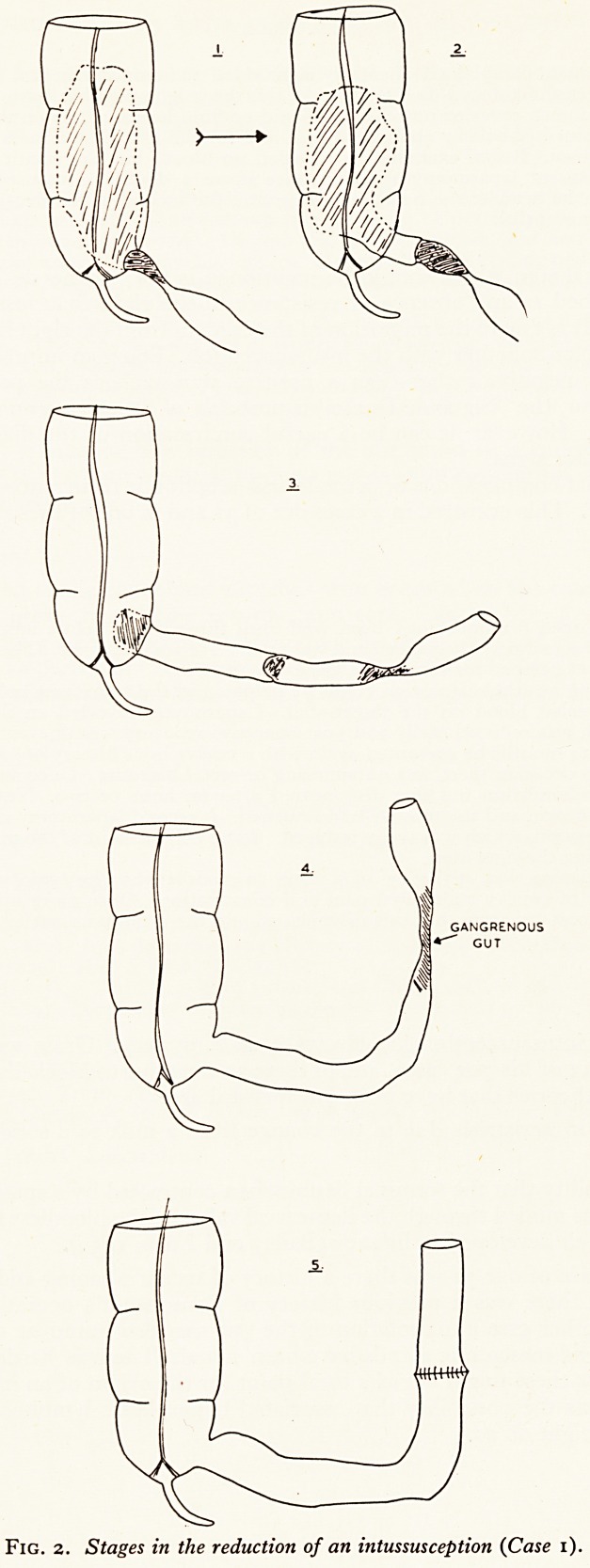# Some Notes on Acute Intussusception in Childhood

**Published:** 1964-04

**Authors:** R. l'E. Orme

**Affiliations:** Department of Paediatrics, Southmead Hospital, Bristol


### SOME NOTES ON ACUTE INTUSSUSCEPTION IN CHILDHOOD

BY

R. l'E. ORME, M.A., M.B., B.CHIR.

*Department of Paediatrics, Southmead Hospital, Bristol*

Sir Zachary Cope (1962) makes it clear in the many editions of his book that intussusception or invagination of the intestine is the most common abdominal emergency in children under the age of 2 years. That this mechanical catastrophe must have presented great difficulty before the days of safe and effective treatment is self-evident from the literature. The following clinical notes describe something of the problems.

### HISTORICAL

As a surgical pathologist John Hunter first described intussusception (Garrison, 1929). Writing in 1829, L. Gaylord related "an extaordinary case of intussusception" as follows.

"A boy aged 6 years presented with abdominal pain, constipation and stercoraceous vomiting. He was treated by bleeding, blistering and the administration of cathartics —first castor oil, then Epsom Salts, jalop, calomel and croton oil. He also had enemata (occasionally of tobacco) and warm baths. The acuteness of the pain, the intensity of the heat together with the accelerated pulse, and the reddened, dark furred tongue and great prostration of strength left scarce a shadow of hope, but he lived on—5 days later evacuation was procured and an alleviation of the alarming symptoms. Two days later a portion of the intestines was protruding and in a state of incipient mortification. A poultice of charcoal and yeast was applied to the part and a decoction of cinchona and brandy administered as the stomach would bear. In one more day the protrusion sloughed off (23 in. in length) but the last 3 in. was contained in a fold which adhered too firmly to be detached, and which had formed the origin and laid the foundation of the original difficulty and obstinacy. This was detached. There followed an imperfect digestion and diminished assimulating powers of nutrition for several weeks. A mercurial course was given to the point of gentle and moderate salivation; health was gradually restored—two years later the boy seemed quite well."

In 1835 W. Thomson described a case in which a portion of the cylinder of the intestinal canal, comprising all its coats was discharged rectally without the continuity of the canal being destroyed. Several singular examples of this "conservatrix naturae" are noted in their course of progression. The cures of nature were accomplished by:

"First the invagination of a portion of intestine, second the detachment by disjunctive absorption of the invaginated from the containing portion of the intestine, and thirdly simultaneously with this disjunctive absorption, the effusion of coagulable lymph around the edges of living intestine from which the separation of the portion about to be discharged is going on, and such an advancement in the process of organisation in this lymph as to give it sufficient firmness to resist the physical impulses to which it may be subjected by the passage of alimentary matters through it. By these singular processes nature effects a cure in circumstances of so threatening a character."

This commentary was written of 35 recovered cases of acute intussusception. The lengths of discharged intestine varied from 6 in. to 40 in. An example of the discharge of a portion of the ileum, caecum and colon was reported by Quain in 1859:

"A boy aged 5 years presented with acute symptoms including vomiting, and was greatly distressed. Four months later he passed the mass of the bowel containing part of the ileum, caecum and colon. The discharge of the bowel was preceded and followed by diarrhoea. The appearance of the faeces became natural and the boy gained perfectly good health".

Another extraordinary situation is revealed in the next story (Fig. I), concerning a boy aged 6.

"This boy passed the caecum with its vermiform process and part of the ascending colon—the mass was passed without the patient's knowledge during deep sleep and next morning he had a natural formed motion. Some weeks before this the boy had signs and symptoms of acute intussusception" (King, 1854).

Attempts to relieve the obstruction by hydrostatic methods were made. Steele (1859) reported a successful result following this procedure. The patient was a girl aged 18 months with an acute intussusception which a gruel enema had failed to relieve.

"A considerable length of inverted gut was occupying the greater part of the rectum. Rectal bougies were unsuccessful against the straining efforts of the child. Persistent treatment was instituted, with the most careful use of a stomach pump to introduce a continuous stream of warm water, at first by slow and gentle actions which were very gradually increased until a fairly full stream was brought to bear and kept up for 10 minutes or more. The water at first gushed back, but at one point of time, the gut no longer descended and all response to the flow eased. The child bore the operation well and some relief of all urgent symptoms. A natural motion was passed 2 days later and the child was quite well some months later."

Dr. Steele hoped that his report in this case would "serve to awaken the vigilance of my brethren and give the stimulus of hope to his labour in the anxious hours of a similar emergency".

Hilton Fagge (1869) had observed that when all the symptoms and signs were fully developed, the fact that a case terminated within 3 or 4 days from the onset was probably in favour of an ileocaecal intussusception, whereas in those cases where the symptoms gradually increase over a period of 10 days or so, the affection was generally of the small intestine, and also that it was in the latter cases that there was a fair hope that the part affected might be thrown off and a cure result. The museum at Guys Hospital at that time contained several specimens of the bowel cast off after intussusception, one of which, from a child of 1 year, was 12 in. long, 1 in. being small intestine and the remainder caecum and ascending colon with the appendix lying within its calibre.

The subject of laparotomy for intussusception was elaborated by Jonathan Hutchinson (1874), and the following quotation reveals the clinical eminence of his paper:

"The patient was a somewhat delicate female child age 2 years. From her anus there protruded a portion of bowel about 2 in. long, deeply congested and much swollen. By the side of this the finger could be passed its full length in to the rectum without reaching the point at which the intussusception began. On carefully examining the extremity of the protruded part, I noticed that it did not present merely a rounded opening as usual in such cases. I was able easily to identify the

FIG. 1

*Drawing of intestinal slough passed during sleep. Case described by C. King in The Lancet of 1854. (Reproduced by kind permission of the Editor, The Lancet.)*

pouch and valve of the caecum, with the opening in to the ileum. Of these parts it was of course the mucous membrane which was visible, and the appendix caeci was wholly concealed between the folds of the intussusception. This discovery rendered it evident that we had to deal with an involution of bowel of very unusual length, which commencing at the caecum had allowed the ileum to pass through the entire length of the colon, and actually to become extruded at the anus."

"The child, at the time of her admission, looked very ill. Her countenance was pale and anxious, and from her mother's description it was evident that her strength had been failing rapidly during the last few days. It appeared that she was almost constantly engaged in straining to get rid of the bowel which filled the rectum. Our first measure of treatment consisted in putting the child under chloroform, and then, whilst she was held up by the feet, distending the rectum to the utmost with warm water."

"By this means the involuted part could be forced up into the abdomen so as to be quite out of reach of the finger, and once or twice I tried to hope that reduction had been effected. On each occasion, however, when the lower bowel was allowed to empty itself, the intussuscepted part became prolapsed as before, and showed clearly that we had gained nothing. It was very evident, from the child's condition, that unless relief was afforded she would not live long, and I therefore felt justified in telling her parents that although an operation would be, in itself, very dangerous yet I thought that it afforded the only chance.

"The child having been taken up into the operation theatre, chloroform was again administered, and I then opened the abdomen in the median line below the umbilicus, and to an extent admitting of the easy introduction of two or three fingers. I now very readily drew out, at the wound, the intussuscepted mass which was about 6 in. long. I found that the serous surfaces did not adhere, and that there was no difficulty whatever in drawing the intussuscepted part out of that into which it had passed. Just as the reduction was finished the appendix caeci made its appearance, confirming the opinion which had been formed as to the precise part of the bowel involved. The opposed serous surfaces did not present a single flake of lymph, and they were congested in only a moderate degree.

"Having completed the reduction, I put the bowel back into the abdomen, and closed the wound with harelip pins and interrupted sutures. The operation had been a simple one and had not occupied more than 2 or 3 minutes.

"No vomiting occurred after the operation. Chloroform was administered on two or three occasions to allow of the wound being dressed without the child's screaming. The pins were taken out on the fourth day, that is, seventy-two hours after the operation. The child recovered without having ever showed the slightest symptom of peritonitis, and left the hospital in excellent health about three weeks after the operation."

Hutchinson clearly set out his carefully considered opinions on the subject of intussusception.

"One fact disclosed by post-mortem records I may ask especial attention to, and that is the almost uniform absence of peritonitis as a complication. This is especially noted in a great number of cases. In intussusception as in strangulated hernia, and other forms of abdominal obstruction, it may, I think, be taken as an established fact, that unless actual perforation has occurred there will be no peritonitis.

"I may briefly record my conviction that any one who will carefully examine the evidence for and against will come to the conclusion that operations for the relief of intussusception are not only warrantable, but that in a large number of cases they are urgently demanded.

"The cases most hopeful are those in which the symptoms denote incarceration rather than strangulation, and in them the surgeon may take the knife in hand with a good prospect that he will encounter no serious obstacle, and that he will not find either very tight constriction, adhesions or gangrene. Of the other cases, there are many in which, if the patient be seen early, there is sufficient hope, notwithstanding the severity of the symptoms, to justify the operation, though the surgeon must expect in such to find occasionally that the conditions preclude its completion. Lastly, in a small minority, seen late, or in which the symptoms have from the first been extremely severe, it is probably wisest to decline an operation and to trust to the chance of gangrene."

In his conclusion we note:

(*a*) That in cases in which the intussuscepted part is incarcerated and not strangulated, there is very little hope of the occurrence of gangrene, and it is probable that the patient will die after some weeks or months, worn out by irritation and pain.

(*b*) That the records of post-mortems justify the belief that, in a considerable portion of the cases referred to, the surgeon will encounter no material difficulty in affecting reduction after opening the abdomen.

(*c*) That the circumstances which might cause difficulty are, first, the tightness of the impaction of the parts; secondly, the existence of adhesions; and thirdly, the presence of gangrene.

(*d*) That in cases of intussusception in young infants (under one year of age) the prognosis is very desperate, scarcely any recovering excepting the few in whom injection treatment is immediately successful, whilst a large majority die very quickly.

(*e*) That the fact just referred to may be held to justify, in that case of young infants very early resort to the operation.

While noting that the vast majority of cases occur in the first year of life, Henoch (1889) thought that it was remarkable for such a tangible condition to have no known cause allocated to it. However, Henoch further observed that:

"There might be a preceding episode of diarrhoea, but that as a rule the disease begins quite suddenly in the midst of perfect health, with violent screaming, great restlessness, frequent vomiting and constipation. Often there occurs also a discharge of a varying quantity of blood from the anus, at first mixed with fragments of faeces, afterwards with mucus and serous fluid, or it may even be discharged pure and partly coagulated.

"The abdomen may retain its normal form and softness for the first 24–28 hours, but then generally becomes tense, distended with flatus, and tender."

Henoch rather astonishingly confesses that he attached no particular importance to the discovery of a tumour, but he was impressed by the occasional ability to feel the rounded end of the intussusception on rectal examination.

"Rarely, the intussusception is extruded from the anus for an inch or so by violent pressing down, and then has the appearance of a dark-red, bloody tumour, with a central opening.

"When we can neither feel the intussusception in the rectum nor see it externally, the diagnosis cannot be made with absolute certainty. It may, however be made with great probability because the combination of these three symptoms—complete obstruction, vomiting, and bleeding from the mucous membrane of the intussuscepted portion of the bowel—are almost conclusive."

There was a clear belief in the possibility of spontaneous resolution of intussusception:

"In cases which end in recovery there is an undoing of the invagination with passage of flatus and faeculent motions; or there may occur separation and discharge of the gangrenous portion of the affected portion of bowel, the normal lumen being restored and the intestine shortened to a corresponding extent."

Whilst searching for a possible line of treatment Henoch hit on the idea that whilst purgatives can only do harm by increasing forward peristalsis, he noted that administration of an "enema of ice-water" might encourage retrograde peristalsis and enable reduction of the intussusception. Occasionally rather more heroic methods of mechanical reduction were practised, such as:

"Inflation of the bowel by means of bellows and the introduction of a whalebone probang with a sponge at the end of it, with which one endeavours to push the invaginated portion of bowel directly upwards when it can be felt in the rectum." Whilst these methods were alleged to be occasionally successful, "The danger lies in the fact that we can never know beforehand whether the intussusception is still reducible, or is already fixed by adhesion of the two serous layers. In the latter case any forcible attempt at reduction might result in the adhesions and even the serous membrane itself giving way—in which case the consequence would of course be fatal."

In the early days of attempts to give surgical relief, desperate cases were subjected to laparotomy under chloroform anaesthesia, but unhappily it was noted that reduction of the intussusception was rarely successful, the reason being that it was nearly always impossible to draw the invaginated portion out of its sheath. The general opinion was held that diagnosis was uncertain in the early stages and strikingly enough that it was impossible to establish any definite indication as to the time when laparotomy was to be performed, and even an intimidating note appeared to the effect that "the Physician who risks this operation must himself, incur responsibility for the result!" There seemed to be a general feeling of pessimism in the words of Henoch in comparison with the more direct belief in the possibilities of successful operative treatment considered, and in fact performed by Hutchinson 15 years earlier.

It is also worthy of emphasis that Gross (1953) still rightly considers that intussusception is one of the most important abdominal emergencies in childhood and some high mortality rates are still being reported. To quote him, "the need for more important knowledge of the condition continues". Undoubtedly, early diagnosis is the governing factor. Fortunately, statistics show favourable trends—a mortality of about 60 per cent in 1910 had changed to a high expectancy of recovery in 1950.

### REVIEW OF CASES

The purpose of this paper is to communicate some facts concerning a review of thirty-one patients in Bristol suffering from intussusception in a period of 10 years. Tables I–III give details of cases under review, Table I relating to uncomplicated cases under one year of age, Table II to similar uncomplicated cases over one year, and Table III (a, b & c) enumerates those cases in which resection of the gut was necessary, in which a double intussusception was found, and finally those cases in which there was a subsequent recurrence.

With regard to age and sex, the figures agree closely with previous papers on the subject. 70 per cent of the children were males and two-thirds of the patients were under one year at first presentation. Of the latter 80 per cent were aged 6 months or less. The eldest patient was a boy of 8 (Case 2). Resection of the gut was necessary in 5 cases and there were no fatalities.

*Uncomplicated cases under 1 year of age.*
*Resection of gut not necessary; no recurrences.*

| Patient | Age | Sex | Time from first symptom to diagnosis | Time from first appearance of blood to diagnosis | Blood in Stools | Vomiting | Abdominal Pain | Tumour | Site* | Co-existent findings |
|---|---|---|---|---|---|---|---|---|---|---|
| V.S. | 4/12 | F | 48 hours | 48 hours | + | + | + | + | I/C | — |
| A.F. | 4/12 | M | 4 days | NIL | + R.C.J. | + | — | + | I/C | — |
| R.K. | 4/12 | M | 12 hours | 9 hours | + | + | + | + | I/C | — |
| G.D. | 5/12 | M | 1 day | 6 hours | + R.C.J. | + | + | + | I/C | — |
| D.F. | 5/12 | F | 12 hours | 3 hours | + | ++ | — | + | I/C | — |
| C.C. | 5/12 | F | 4 hours | 4 hours | + R.C.J. | ++ | — | — | I/C | 1. Previous pyloric stenosis. 2. Cereal diet. |
| E.N. | 5/12 | F | 6 hours | NIL | + R.C.J. | + | + | + | I/C | Recent triple vaccination. |
| R.L. | 5/12 | M | 2 days | 12 hours | + | ++ | + | + | I/C | — |
| P.T. | 5/12 | M | 3 hours | — | — | + | + | + | I/C | Mesenteric glands + |
| T.W. | 6/12 | M | 3 hours | NIL | + R.C.J. | + | + | + | I/C | — |
| M.G. | 7/12 | M | 34 hours | 24 hours | ++ | + | + | + | I/C | — |
| C.B. | 8/12 | M | 12 hours | 6 hours | + | ++ | + | + | I/C | Mesenteric glands + |
| G.D. | 9/12 | M | 36 hours | 5 hours | + | + | + | + | I/C | — |
| R.L. | 2/12 | M | 48 hours | NIL | + R.C.J. | ++ | + | + | I/C | Malrotation of gut. |
| M.C. | 2/12 | M | 4 days | 1 day | + | ++ | — | + | I/I | — |

* I = Ileum, C = Colon, J = jejunum.

TABLE II

Uncomplicated cases over 1 year of age.
Resection of gut not necessary; no recurrences.

| Patient | Age | Sex | Time from first symptom to diagnosis | Time from first appearance of blood to diagnosis | Blood in Stools | Vomiting | Abdominal Pain | Tumour | Site* | Co-existent findings |
|---|---|---|---|---|---|---|---|---|---|---|
| R.D. | 14/12 | M | 5 hours | 5 hours | + | + | — | + | I/C | Mesenteric glands |
| M.R. | 18/12 | M | 12 hours | 3 hours | + | + | — | + | C/C | — |
| P.B. | 18/12 | M | 3 hours | | — | + | + | + | I/C | Mesenteric glands |
| N.C. | 18/12 | M | 5 hours | 2 hours | + | + | + | + | I/C | — |
| M.R. | 24/12 | M | 6 days | | Occult + | ++ | ++ | + | I/C | — |
| C.R. | 29/12 | M | 6 hours | NIL | + R.C.J. | ++ | + | + | I/C | — |
| G.M. | 7 | F | 15 hours | NIL | + | + | + | + | C/C | — |

* I = Ileum, C = Colon, J = jejunum.

TABLE III (*a*, *b*, *c*)

*(a) Cases requiring gut resection. (b) Double intussusceptions. (c) Cases where there was a subsequent recurrence.*

| Patient | Age | Sex | Time from first symptom to diagnosis | Time from first appearance of blood to diagnosis | Blood in Stools | Vomiting | Abdominal Pain | Tumour | Site* | Co-existent findings. |
|---|---|---|---|---|---|---|---|---|---|---|
| (*a*) R.G. | 8 | M | 3 days | | Occult + | — | ++ | + | 1. I/C<br>2. I/J | — |
| L.R. | 11/12 | F | 18 hours | | — | + | + | + | 1. I/C<br>2. C/C | — |
| J.C. | 6/12 | M | 2 days | NIL | + | + | + | + | 1. I/C<br>2. I/I | — |
| (*b*) J.C. | 6/12 | M | 2 days | NIL | + | + | + | + | 1. I/C<br>2. I I | — |
| T.K. | 5/12 | M | 4 days | NIL | + | + | + | — | I/4 | — |
| J.H. | 5/12 | F | 3 days | 5 hours | R.C.J. | + | — | + | I I | — |
| J.N. | 13/12 | F | 3 days | | — | + | — | + | I/I | — |
| M.S. | 3 | M | 4 attacks in 17/12 lasting 2–3 days | | + | + | + | — | I/C | Mesenteric glands |
| (*c*) M.D. | 6/12 | M | 3 hours | | + | + | + | + | I/C | — |
| M.D. | 14 12 | M | 12 hours | | — | — | + | + | I/C | Mesenteric glands |
| M.D. | 2 10 12 | M | 6 hours | | — | — | + | + | Unknown | — |
| V.H. | 4 | F | 3 days | | — | + | + | — | I/C | — |
| V.H. | 5½ | F | 1 day | | — | — | + | — | I/C | Mesenteric glands |

* I = Ileum, C = Colon, J = jejunum.

### DIAGNOSIS

Early diagnosis is of particular importance in this condition, in that if the necessity for resection of the intussusception can be avoided the prognosis is much improved. Gross (1953) referring to a series prior to 1939 reported no deaths at all in 110 cases where the diagnosis was made within twenty-four hours of the onset of symptoms. In cases where the delay was longer than twenty-four hours there were 27 deaths in 92 cases. Fortunately modern surgical techniques have greatly improved the prognosis in the latter group. Of the 5 cases under discussion in which resection was necessary the time between onset and diagnosis averaged seventy-two hours (range 48–96 hours).

The symptoms most suggestive of acute intussusception are the triad of:

1. Colicky abdominal pain.
2. Vomiting.
3. The passage of blood in the motions.

Of these the passage of blood in the motions is undoubtedly the most specific and probably that most depended on in making the diagnosis. However, although the most specific, it is also by far the most inconstant. In our series frank blood in the stools was only noted in a little over 60 per cent of cases, there was no blood in the stools at any time in 26 per cent of cases, and in 12 per cent blood was only demonstrable by rectal examination or by chemical tests. In only 20 per cent were the stools described as being of the classical red-currant jelly appearance.

It should also be noted that often blood does not appear in the stools until a considerable period after the onset, often only after several days. The longest period I have found was, in fact, 4 days (Case 4). In cases of this sort the intussuscepted bowel may have already become gangrenous.

*Case 1*

J. C. aged 6 months presented with a history of having been lethargic for 2 days and having vomited all his feeds, even boiled water, In between feeds he had slept, but every few minutes he had awakened crying, after which he slowly settled once more. The bowels had not been open for the same period.

On admission he was extremely dehydrated. The abdomen was very distended, with clearly visible distended loops of small bowel. The head of the intussusception could be felt at the splenic flexture, and on removing the nappy it was found for the first time to be streaked with blood. Rectal examination revealed red-currant jelly stools.

He was treated with intravenous fluids and later the same day a laporotomy was performed. A double intussusception was found (Fig. 2) which was reduced with difficulty. The head of the intussusception was found to be gangrenous and 2 cm. of ileum had to be resected. Post-operative recovery was uneventful. The child was discharged 3 weeks later quite well.

Far more important early symptoms are the persistent vomiting which was present in nearly 90 per cent of cases, and the recurrent colicky abdominal pain (75 per cent of cases). In children who were too young to describe their symptoms this was usually apparent by the recurrent bouts of screaming as described in the case above.

The most important positive clinical finding is the palpation of the head of the intussusceptum as a firm tumour in the line of the colon. Occasionally it may only be felt rectally. A case in which this was the only positive finding on which the diagnosis could be made is described below.

*Case 2*

R. G. aged 8 presented with a history of colicky abdominal pain for twenty-four hours.

On examination on admission he was afebrile and pain-free, and there were no abnormal physical signs. The urine was cloudy and on microscopy contained scanty pus cells only.

He was kept under observation and slept well throughout the night.

FIG. 2. *Stages in the reduction of an intussusception (Case 1).*

Next day he again complained of colicky right sided abdominal pain and was found to be tender over the right kidney. It was thought that he might possibly have renal colic, but X-ray of the abdomen revealed no renal stone and no fluid levels. Early on the following day he again complained of colicky abdominal pain and for the first time a mass was palpable in the right iliac fossa. Rectal examination revealed no blood, but the occult blood test was positive. Subsequent laparotomy revealed once more a double intussusception, the first ileo-caecal, with its head at the hepatic flexture and the second at the ileo-jejunal junction. Recovery was uneventful.

Another clinical sign which should be mentioned is the "Signe de Danse". This has been described as an "absence of resistance in the right iliac fossa" (Rose and Carless, 1924). It is due to the migration of the caecum from the right iliac fossa along the line of the colon together with the intussusception. Being an impression of something essentially negative rather than a positive sign such as the palpation of the abdominal tumour, the "Signe de Danse" is probably of little value on its own except in expert hands. However, it can be a useful confirmation of the diagnosis in conjunction with other signs.

One of the chief complications of acute intussusception is recurrence of the invagination of the gut. This occurred in 2 cases out of 31 and in one of these the recurrence occurred twice.

*Case* 3

M. D. a boy aged 6 months had been well until three hours before admission when he suddenly started screaming after a feed and went pale. He vomited once. The stools had been dark, but this had been so for some time since weaning.

On examination an intussusception could be palpated in the transverse colon, and a rectal examination revealed blood on the finger-stall. Laparotomy revealed an ileo-caecal intussusception which was reduced easily and post-operative recovery was uneventful.

At the age of 14 months he presented again with a twelve hour history of colicky abdominal pain, but on this occasion there was no vomiting or rectal bleeding. Once again a mass was felt in the hypochondrium but this disappeared after an hour or two. Next day he again appeared to be in pain and the tumour had returned. A second laparotomy revealed an ileo-caecal intussusception, which was easily reduced. Some enlargement of the mesenteric glands were found during the operation.

His third admission was at the age of 2 years 10 months when he again presented with a six hour history of colicky abdominal pain and constipation. Once more a tumour was felt under the right costal margin, but this disappeared and the symptoms settled without further operation.

### CAUSE

The cause of intussusception has always been a mystery. Gross was able to find a cause in only 43 of his 702 cases, and of these 32 were due to Meckel's diverticulum. Amongst other theories that have been put forward are:

1. Alteration in peristalsis due to the change from a milk to a solid diet. (Gross, 1953.)
2. The possibility that the terminal ileum when contracted by a spasm of intestinal colic may be guided through the ileo-caecal valve by the bloodless fold of Treves, which is well developed in infancy (Bailey and Love, 1959).

In only one case of our 31 was there a history of recent weaning and that case was peculiar in that there was a previous history of Rammstedt's operation for pyloric stenosis. In another case a malrotation of the gut was also found at operation, and in 7 cases enlarged mesenteric glands have been noted. There is hardly enough evidence to state that these might act as a focal point for the origin of an intussusception, but there remains the possibility that associated hyperplastic lymphoid tissue in the intestinal wall might do so.

One further case must be mentioned, in that even in retrospect early diagnosis would have needed even more than the "unusual acumen and luck" that has been recommended by a contemporary magazine.

*Case 4*

A boy aged 5 months was admitted having been well until 4 days previously when he became unwilling to take his feeds. With perseverance he could be persuaded to take half his feeds, but would often vomit afterwards. He had also been miserable, and had been crying a lot, but had not been noticed to draw his legs up. He had also been constipated, but there were no other symptoms. Not until the fourth day of his illness had he passed blood rectally, when he passed a quantity of altered blood.

On examination he looked extremely well. He was well nourished and there were no signs of dehydration. The abdomen was not distended, there were no signs of obstruction clinically and no tumour was palpable. At operation an ileo-ileal intussusception was found which was gangrenous at the point of origin. 13 cm. of ileum had to be resected. Post-operative recovery was slow but uneventful, and he was discharged fit and well.

It is difficult to imagine how resection of the gut could be avoided in such a case, short of going to the extreme of subjecting every child presenting with an attack of vomiting to a barium enema.

*Acknowledgement*

I am indebted to the consultant surgeons who operated on the cases mentioned for permission to quote from their records, and especially to Mr. A. G. McPherson, F.R.C.S., who also gave permission to reproduce his diagrams; and to Mr. Sweet of the Photographic Department, Southmead Hospital. I should also like to express my gratitude to Professor A. V. Neale for his great help and guidance in the preparation of this paper.

REFERENCES

Bailey, H. H., and Love, R. J. M. (1959). "A short Practice of Surgery", 11th Edition. Lewis, London.

Cope, Sir Zachary (1962). "The Early Diagnosis of the Acute Abdomen", 12th Edition. Oxford University Press.

Fagge, Hilton (1869). Guy's Hosp. Reports, **14**, 272.

Garrison, L. (1929). Introduction to the History of Medicine "John Hunter", 4th Edition.

Gaylord, L. (1929). *Am. J. Med. Sci.*, **5**, 318.

Gross, R. E. (1953). "The Surgery of Infancy and Childhood", p. 281.

Henoch, E. (1889). Lectures of Children's Diseases (translation) pp. 66–70. New Sydenham Society of London.

Hutchinson, J. (1874). Medico-Chirurgical Transactions of London. Series 2, **39**, 31.

King, C. (1854). *Lancet*, **i**, 368.

Quain, R. (1859). *Trans. Path. Soc. London*, **10**, 160.

Rose, W., and Carless, A. (1924). Manual of Surgery, 11th Edition. Baillière, Tindall, and Cox.

Steele, E. Y. (1859). *Lancet*, **i**, 28.

## Figures and Tables

**Fig. 1 f1:**
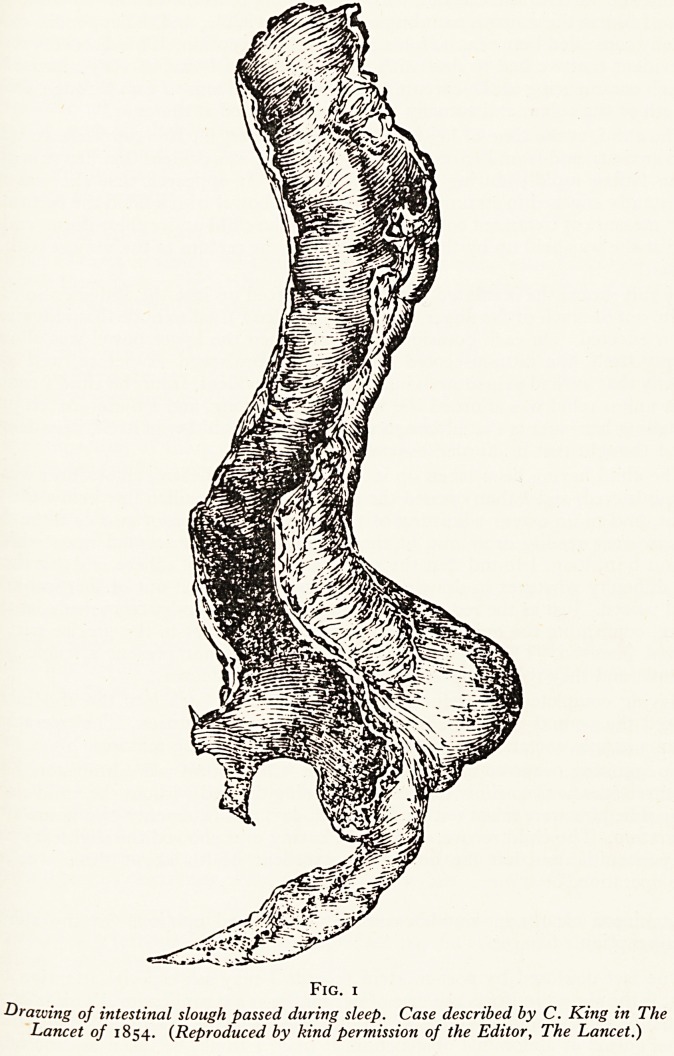


**Fig. 2. f2:**